# Learning the kernel for rare variant genetic association test

**DOI:** 10.3389/fgene.2023.1245238

**Published:** 2023-10-09

**Authors:** Isak Falk, Millie Zhao, Juba Nait Saada, Qi Guo

**Affiliations:** ^1^ Department of Computer Science, University College London, London, United Kingdom; ^2^ Computational Statistics and Machine Learning, Italian Institute of Technology, Genoa, Italy; ^3^ BenevolentAI, London, United Kingdom

**Keywords:** GWAS, kernel learning, reproducing kernel Hilbert space, score testing, SKAT, target alignment, WES

## Abstract

**Introduction:** Compared to Genome-Wide Association Studies (GWAS) for common variants, single-marker association analysis for rare variants is underpowered. Set-based association analyses for rare variants are powerful tools that capture some of the missing heritability in trait association studies.

**Methods:** We extend the convex-optimized SKAT (cSKAT) test set procedure which learns from data the optimal convex combination of kernels, to the full Generalised Linear Model (GLM) setting with arbitrary non-genetic covariates. We call this extended cSKAT (ecSKAT) and show that the resulting optimization problem is a quadratic programming problem that can be solved with no additional cost compared to cSKAT.

**Results:** We show that a modified objective is related to an upper bound for the *p*-value through a decreasing exponential term in the objective function, indicating that optimizing this objective function is a principled way of learning the combination of kernels. We evaluate the performance of the proposed method on continuous and binary traits using simulation studies and illustrate its application using UK Biobank Whole Exome Sequencing data on hand grip strength and systemic lupus erythematosus rare variant association analysis.

**Discussion:** Our proposed ecSKAT method enables correcting for important confounders in association studies such as age, sex or population structure for both quantitative and binary traits. Simulation studies showed that ecSKAT can recover sensible weights and achieve higher power across different sample sizes and misspecification settings. Compared to the burden test and SKAT method, ecSKAT gives a lower *p*-value for the genes tested in both quantitative and binary traits in the UKBiobank cohort.

## 1 Introduction

Genome-wide association studies (GWAS) (Visscher et al., 2017) have been shown to be a powerful way to identify common genetic variants (Cordell and Clayton, 2005); (Hindorff et al., 2009). However, for most diseases, the common susceptibility variants identified to date explain only a small proportion of the heritable component of disease risk. It is known that for low-frequency variants and rare variants, the power to detect the effect is limited (Lee et al., 2014) in GWAS. Rare variants are known to play an important role in human diseases. It is a well-established hypothesis that rare variants may be able to explain the missing heritability (Zuk et al., 2014).

The common approach to tackle this problem is to aggregate the variants in a gene set. Most of these testing procedures can be classified into two groups: the burden test, which collapses the SNPs in the gene set into one scalar value to then be regressed onto the trait (Morgenthaler and Thilly, 2007); (Lee et al., 2014); (Guo et al., 2016), and variance component tests, of which the sequence kernel association test (SKAT) is the prototypical procedure in genetic testing (Wu et al., 2011; Lee et al., 2012; Liu et al., 2019). SKAT is more flexible than the burden test because it makes fewer assumptions about the data, but the burden test has greater power when a large proportion of variants are causal and effects are in the same direction. In reality, the burden test can be shown to be a special case of SKAT (Wu et al., 2011), and the unifying framework is that of kernels (Aronszajn, 1950; Hofmann et al., 2008). The kernel framework allows for building a kernel, encoding affinity between two objects such as gene sets (Borgwardt, 2011). However, with the current availability of data, it is possible to learn a superior kernel from the data itself, commonly known as learning the kernel or multiple kernel learning (Cortes et al., 2012; Gönen and Alpaydın, 2011; Sonnenburg et al., 2006). Posner et al. (2020) proposed a way to learn a kernel as a convex combination of linear kernels from the data itself, calling this procedure convex SKAT (cSKAT). Although Posner et al. (2020) claimed to handle non-genetic covariates beyond the regression setting, their derivation is flawed due to a mistake, leading to the wrong denominator in the objective (Supplementary Appendix SA1). Due to this flaw, their method (cSKAT) only optimizes the correct objective for the regression setting with no non-genetic covariates and does not extend to the case when we have non-genetic covariates, which is important for accounting for population stratification (Cardon and Palmer, 2003), for potentially binary traits.

In this paper, we extend cSKAT (Posner et al., 2020) to any statistical model that comes under the mixed linear model (Zhang et al., 2010) (an extension of the generalized linear model (GLM) framework Nelder and Wedderburn (1972), which generalizes regression to a large family of models, including logistic regression) while allowing for non-genetic covariates. We call the resulting model extended cSKAT (ecSKAT). We note that although Posner et al. (2020) focused on annotated genetic data, their method applies beyond this setting (Section 2.4), which shows the process in detail. Although ecSKAT can be applied to annotated genetic data, we do not pursue this direction here and instead focus on the standard genetic data included in our experiments. The model has several advantages over standard SKAT because it allows for learning the kernel in a data-driven way by solving an optimization problem shown to be equivalent to a quadratic program (QP), leading to increased power over using a hand-picked kernel. Hence, it has the same computational and memory complexity as that of Posner et al. (2020), so the additional generality comes with no extra cost. Theoretically, we use concentration inequalities for a convex combination of independent *χ*2-random variables to show that a modified ecSKAT objective relates to the null *p*-value through an upper bound that reaches zero exponentially in the objective value.

## 2 Materials and methods

We are interested in hypothesis testing using the score test in the context of genomic studies; in particular, given a dataset of patients with a response, non-genetic covariates, and a number of gene sets, for each gene set, we are interested in testing for the association between the gene set and response, taking into account the non-genetic covariates. Our work extends cSKAT (Posner et al., 2020), which itself comes from the line of work initiated by Wu et al. (2011). Interestingly, both Lee et al. (2012) and Ionita-Laza et al. (2013) proposed a convex combination of two very specific kernels, but the papers did not make this connection explicitly, which was pointed out in Larson et al. (2019). Zhao et al. (2015) created an algorithm for learning a kernel from multiple base kernels, which is probably the work closest to our proposed method, except for cSKAT. However, their algorithm is expensive as it requires us to calculate *p*-values for each iteration, while ours is efficient due to the optimization step being QP.

### 2.1 Model

We consider a genetic association study of sample size *n* where we try to find a significant association between genetic variants and some outcomes (a trait or phenotype generally associated with disease) while controlling for stratification by taking into account non-genetic covariates. Given non-genetic and genetic covariates denoted by $x\in R^{m}$ and *g* ∈ {0,1,2}^*p*^ ^1^, respectively, and output y∈R from *n* subjects, where *m* and *p* are the non-genetic and genetic dimensions, we collect the data into a dataset $D={\left(x_{i},g_{i},y_{i}\right)}_{i=1}^{n}$ . Additionally, we define the non-genetic and genetic design matrices $X\in R^{n\times m}$ and $G\in R^{n\times p}$ , respectively, and the output vector $y\in R^{n}$ . For logistic regression, we encode an outcome as 1 and a lack of outcomes as 0. We limit ourselves to the setting of linear and logistic regression but note that other models, such as multinomial and Poisson regression, are easily handled due to the flexibility of the GLM framework (Nelder and Wedderburn, 1972), and the model we use for the testing procedure is that of the generalized linear mixed-effect model (Gelman and Hill, 2006), relating the phenotype to the genetic and non-genetic covariates.

For a gene set giving rise to the genetic design matrix ***G***, we are interested in testing for an association between the trait and genetic information. We use the standard frequentist hypothesis testing framework (Casella and Berger, 2021), and we formulate the null and alternative hypotheses as follows:

$$H_{0}:h=0,\;H_{alt}:h\neq 0.$$ (1)

Letting *h* to be linear in some set of parameters $\beta \in R^{p}$ , then the function space containing *h* consists of linear functions of the form $h\left(g\right)=\beta^{T}g$ with some inner products defined between functions $h=\beta^{T}g,h^{\prime }\left(x\right)=\beta^{\prime T}g$ , and $\left\langle h,h^{\prime }\right\rangle =\beta^{T}\Lambda \beta^{\prime }$ for some positive semi-definite **Λ**. If **Λ** is full-rank, Eq. 1 becomes

$$H_{0}:\beta =0,\;H_{alt}:\beta \neq 0.$$ (2)

### 2.2 Score tests

SKAT was first introduced in Wu et al. (2011), who highlighted the need for new association tests that may take into account the importance of rare variants while being able to incorporate genetic effects that are sparse and have different directions of impact on the response, such as some genetic variants being deleterious while others being beneficial. The burden test (Madsen and Browning, 2009), another commonly used testing procedure, typically struggles with these kinds of settings. SKAT uses the variance component score test (Lin, 1997) to devise a testing procedure that takes into account effect sizes of differing signs and allows injecting prior knowledge about how rarity is related to the magnitude of effect size.

The family of SKAT-like testing procedures, including their many descendants (Lee et al., 2012; 2014) (Larson et al., 2019), assume that *β* follows Gaussian distribution, which makes it explicitly linked to Gaussian processes (Rasmussen, 2003), and something that was also pointed out in Wu et al. (2011). In particular, assuming that *h* follows a Gaussian process with mean function 0 and covariance function *τK*(⋅, ⋅) with *τ* ≥ 0 and *K* a valid kernel function, the vectorized output $h\left(G\right)={\left(h\left(g_{i}\right)\right)}_{i=1}^{n}$ follows the Gaussian distribution *N*(0, *τ* ***K***)^2^, where ***K*** *ij* = *K*(*g_i_*, *g_j_*) is the so-called kernel matrix encoding affinity between the individuals through the gene set. When $h\left(g\right)=\beta^{T}g$ , this is equivalent to *β* ∼ *N*(0, *τ* ⋅ diag(***w***))^3^ for some ***w*,** where we assume ***w*** ∈ △_*p*_, with △_*p*_ being the set of probability vectors of dimension *p* ^4^, for simplicity. Choosing ***w*** allows us to design a test that fits the domain knowledge by weighing the contribution of individual variants according to the weight vector ***w***. From here on, we will only consider weighted linear kernels $K\left(g,g^{\prime }\right)=\sum_{j=1}^{p}w_{j}g_{j}g_{j}^{\prime }$, where ***w*** ∈ △_*p*_.

In this case, we can reformulate Eq. 1 as an equivalent to testing if

$$H_{0}:\tau =0,\;H_{alt}:\tau >0,$$ (3)

and the variance component test (Lin, 1997), in this case, reduces to the SKAT statistic (Wu et al., 2011). Let $\left({\hat{\alpha }}_{0},\hat{\alpha }\right)=\hat{\tilde{\alpha }}$ and ${\hat{\mu }}_{0}=\eta^{-1}\left({\hat{\alpha }}_{0}+X\hat{\alpha }\right)$ be the maximum likelihood conditional mean and $r=y-{\hat{\mu }}_{0}$ be the raw residuals of the null model. Then, the SKAT statistic takes the following form:

$$Q_{SKAT}=\frac{r^{T}Kr}{{\hat{\phi }}_{0}},$$ (4)

where ${\hat{\phi }}_{0}$ is the maximum likelihood estimate of the dispersion parameter. For the case of a binary outcome, ${\hat{\phi }}_{0}=1$ and for the continuous outcome ${\hat{\phi }}_{0}={\hat{\sigma }}_{0}^{2}$, which is the residual sample variance under the null model. Under the null hypothesis, the asymptotic distribution of $Q_{SKAT}$ follows a mixture of independent $\chi_{1}^{2}$ variables with the mixture coefficients being the eigenvalues of the matrix $A=P_{0}^{1/2}KP_{0}^{1/2}/{\hat{\phi }}_{0}$ , where $P_{0}=V-VX{\left(X^{T}VX\right)}^{-1}X^{T}V$ , ***P***_0_ is a positive semi-definite, and ***V*** = diag(***v***), where ***v***_*i*_ is the maximum likelihood conditional variance of *y_i_* under the null model. For binary outcomes, $v_{i}=\frac{1}{{\hat{\mu }}_{0}\left(1-{\hat{\mu }}_{0}\right)}$ , while for continuous outcomes, it takes the form $v_{i}={\hat{\sigma }}_{0}^{2}$ (Wu et al., 2011; Posner et al., 2020). We let ***λ***_0_ be the eigenvalues of ***A***, denoted by ***λ***_0_ = eig(***A***).

Based on the aforementioned findings, we obtain the *p*-value function as follows:

$$p_{0}\left(q\right)=\mathrm{Pr}\left(Q_{SKAT}\geq q\right),$$ (5)

where $Q_{SKAT}=\sum_{j=1}^{p}\lambda_{0,j}\chi_{1}^{2}$ and the probability is with respect to the null distribution. We cannot evaluate p_0_ analytically, but it can be evaluated numerically up to arbitrary precision using Davie’s method (Davies, 1980) or approximately (Liu et al., 2009).

### 2.3 Extended convex-optimized SKAT

In this section, we extend the analysis of Posner et al. (2020) as follows: first, we show that their annotated kernel formulation can be reformulated as a specific kernel through an explicit feature map of *g* and we generalize this formulation. Second, we derive the objective in case of the existence of non-genetic covariates and models other than linear regression and show that this leads to an objective that results in a similar but qualitatively different solution compared to centered kernel target alignment, which can nevertheless be solved efficiently through a QP. Finally, using a large deviation theory, we show that the objective proposed in Posner et al. (2020) is related to an *upper bound* on the *p*-value under the null hypothesis as maximizing the objective minimizes the upper bound, putting the proposed solution on a principled footing and clarifying the nature of the weights that ecSKAT learns.

### 2.4 Reducing cSKAT to ecSKAT

Although Posner et al. (2020) introduced their method in the setting of learning with genetic annotations, we show, in this section, that their method is a multiple kernel learning method in disguise and so can be applied to any setting where we have a dataset in the form of an input matrix ***G***, confounding input matrix ***X*** and traits ***y***, where ***G*** can now be any matrix of stacked feature vectors per patient which we want to relate to traits ***y***. In particular, ***G*** can be the original genetic design matrix or the aggregated genetic annotation matrix $\tilde{G}$ defined as follows.

Posner et al. (2020) assumed that for each SNP indexed by *j*, we have a sequence of annotation vectors ${\left(a_{l,j}\right)}_{l=1}^{L}\in R^{d_{1}}\times \cdots \times R^{d_{L}}$ , where *d_l_* is the dimensionality of ***a***_*l*,*j*_ for any *j*. The form of the kernel they proposed is $K_{w}\left(g,g^{\prime }\right)=\sum_{l=1}^{L}w_{l}K_{l}\left(g,g^{\prime }\right)$ , where ***w*** ∈ △_*L*_ and $K_{l}\left(g,g^{\prime }\right)\propto \sum_{j=1}^{p}{\left(1^{T}a_{l,j}\right)}^{2}g_{j}g_{j}^{\prime }$ . It should be noticed that ${\left(1^{T}a_{l,j}\right)}^{2}$ is a scalar function of ***a***_*l*,*j*_, so replacing this by any scalar function $\phi_{l}:R^{d_{l}}\to R_{+}$ , where we enforce positivity to make sure that *K_l_* is a valid kernel, does not change the form of *K_l_*. This would allow for further flexibility in choosing how to aggregate annotation data, if available, with some suggestions being $\phi_{l}\left(x\right)={\left|x\right|}^{p}$ for *p* ≥ 1 or *ϕ_l_* (*x*) = exp(−*x*), which leads to the so-called softmax weighing function. In practice, one could choose *ϕ_l_* from a pool of candidates (for example, from those outlined previously) using cross-validation on the train set ((Hastie et al., 2009; Section 7.10) for an introduction to cross-validation). For simplicity, we can assume from here that *ϕ_l_* (*x*) = *x* ^2^, which reduces to using the aggregation method in Posner et al. (2020). Let $\Phi_{l}\in R^{p}$ be the vector such that Φ_*l*,*j*_ = *ϕ_l_* (***a***_*l*,*j*_), ***D***_*l*_ = diag(Φ_*l*_), and $F=\left[\sqrt{D_{1}},\ldots ,\sqrt{D_{L}}\right]\in R^{L\times p}$ . Then, let the transformed genetic vector be $\tilde{g}=Fg$ and define $\tilde{G}$ to be the design matrix of this new genetic dataset of the cohort. The kernel matrix can then be expressed in the form $K=\tilde{G}W{\tilde{G}}^{T}=GF^{T}WFG^{T}$ , which is of the form we considered previously. This shows that the setting of Posner et al. (2020) can be handled in the general linear kernel case where the genetic feature vectors are first preprocessed using ***F***, and ***w*** is then learned using multiple kernel learning techniques (Cortes et al., 2012). In this work, we do not use annotations for simplicity and only consider $K=G^{T}WG$ .

### 2.5 General cSKAT

Posner et al. (2020) laid out a strategy for how to select ***w*** in a data-driven way but only derived an explicit form for the case of no non-genetic covariates (only fitting *α*_0_, which can be shown to be equivalent to centering ***y*** in this case) and linear regression. Here, we solve the full case when ***X*** is non-zero and for any valid conditional response model that comes under the GLM framework with a canonical link function. They proposed to split the data into a train and a validation set, *D* = *D*_tr_ ∪ *D*_ts_, where *D*_tr_ is used to finding ***w*** and *D*_ts_ to perform the hypothesis test using ***w***. The learning procedure of ***w*** is defined through the following objective:

$$J\left(w\right)=\frac{Q_{SKAT}\left(w\right)}{\|{\lambda_{0}\left(w\right)\|}_{2}},$$ (6)

where we view $Q_{SKAT}$ and ***λ***_0_ as functions of *K* **_*w*_** through ***w***. The induced optimization problem becomes

$$w^{*}=\underset{w\in {△}_{p}}{\mathrm{arg max}}J\left(w\right).$$ (7)

As shown in theorem 1.1, we may rewrite $J\left(w\right)=\frac{w^{T}s}{\|{w\|}_{B⊙B}}$ , where $B=G^{T}\left(V-VX{\left(X^{T}VX\right)}^{-1}X^{T}V\right)G$ and $s={\left(G^{T}r\right)}^{2}$ is the component score vector where the square is applied component-wise, and the solution ***w*** * in Eq. 7 can be shown to be proportional to the solution of QP:

$$w^{*}\propto \underset{z\geq 0}{\mathrm{arg min}}z^{T}\left(B⊙B\right)z-2z^{T}s,$$ (8)

which can be solved effectively using modern convex solvers (Diamond and Boyd, 2016).

### 2.6 Relating optimization objective to *p*-value

As pointed out in Posner et al. (2020, A1), there is no a-priori reason that optimizing the objective (Eq. 6) will lead to a test with good power, what we would like to do theoretically is to maximize the power directly. As a proxy to power maximization, we would instead prefer minimizing the *p*-value (Eq. 5) on the training set in terms of ***w***. However, it is not clear how to optimize the *p*-value since it is highly non-convex and complicated. A commonly used approach in optimization is to instead optimize an upper bound, p_0_(*Q*(***w***); ***w***) ≤ *u*(***w***), where *u*(***w***) is tight and convex. Here, we have explained explicitly the dependency of p_0_(*q*) on ***w***.

In theorem 1.2, we show using large deviation theory (Wainwright, 2019; Vershynin, 2018) in the form of sub-exponential concentration inequalities applied to the linear combination of independent $\chi_{1}^{2}$ random variables that the *p*-value is upper-bounded through

$$p_{0}\left(Q\left(w\right)\right)\leq \exp\left.\left(-\frac{1}{8}\min\left(J\left(w\right),J{\left(w\right)}^{2}\right)\right)\right.,$$ (9)

where *J*(***w***) differs from the cSKAT objective (Eq. 7) as the numerator now takes the form $w^{T}\left(s-b\right)$ , where ***b*** = diag(***B***), the diagonal of ***B*** as a vector, instead of $w^{T}s$ . Assuming that *J*(***w***) ≥ 1, then the upper bound is exp(−*J*(***w***)/8). Since the function *f*(*x*) = exp(−*x*/8) is decreasing, we observe that minimizing *f* is equivalent to maximizing *J*(***w***), which again reduces to a QP problem similar to Eq. 22. The result shows that the modified cSKAT objective is a principled objective and that maximizing it is equivalent to minimizing an upper bound on the *p*-value, and furthermore, as the objective grows in value, the upper bound decreases and reaches zero as *J*(***w***) → *∞* exponentially fast. In particular, for any *a* ≥ 0, fixing a minimum level p_0_(*Q*(***w***); ***w***) ≤ 10^−*a*^ can be certified as long as $a\geq \frac{1}{8}\min\left(J\left(w\right),J{\left(w\right)}^{2}\right)$ , which is easy to check after optimization.

## 3 Results

### 3.1 Synthetic and semi-empirical models

In order to benchmark the models (including ecSKAT), we need to know how the genetic and non-genetic covariates are related to the output. We simulate the data using a model of the relationship of the GLM form as follows:

$$\eta \left(\mu \left(x,g\right)\right)=\alpha_{0}+\alpha^{T}x+\beta^{T}g+g^{T}\Gamma g,$$ (10)

where we generate *α*_0_, *α* and *β* together with a potential genetic interaction term $g^{T}\Gamma g$ , where Γ has zero diagonal and is only non-zero for the causal terms corresponding to *β*. The interaction term is only used when evaluating the performance of the model under *model misspecification* and Γ = 0 when there is no model misspecification. Model misspecification aims to answer the question of what happens when using a model that specifies some assumptions of the world when these assumptions are violated in some pre-specified way. In this case, we aim to capture what happens when the model is violated in the sense that the linear term $\beta^{T}g$ is replaced by the linear and interaction term $\beta^{T}g+g^{T}\Gamma g={\left(\beta^{T}+\Gamma g\right)}^{T}g$ . As in practice, our model is always misspecified, seeing how the methods performing under misspecification is integral, and we would prefer the procedure to degrade gracefully when there is non-severe model misspecification. The following settings depend on the marginal distribution and functional relationship, among others (Supplementary Appendix SA1), for a detailed specification for each setting.

We benchmark ecSKAT against the burden test using the sum as an aggregation, uniform SKAT, where the weights are equal to 1/*p*, and SKAT, where we set the weights using *β*(1, 25)-pdf, as outlined in Wu et al. (2011); we denote these algorithms by ecSKAT, Burden (Sum), SKAT (Uniform), and SKAT (*β*(1, 25)) in the figures and experiments, in terms of estimated Type I error and power. For the marginal distributions of the non-genetic and genetic covariates (*ρ_X_* and *ρ_G_*), we either generate them synthetically or use the empirical distributions of the UKBiobank dataset through using available datapoints (patients) relating to the gene PARK7 with non-genetic covariates of age (in years), sex (one-hot encoded), and 10 principal components from the full genetic whole-exome sequencing (WES) dataset and sample without replacement (due to the size of the database the id violation is negligible) from these patients 1,000 times in order to get 95% confidence intervals for Figures 3, 4. For ecSKAT, we use a train ratio of 0.3. Another approach would be to consider the marginal distribution of evolutionary simulation models such as in Wu et al. (2011), Hamilton (2021), and Yuan et al. (2012). We instead use the empirical data directly as they explain the ground truth and large sample size in UKBiobank to make this feasible without introducing artifacts due to the finite size of the original dataset.

**FIGURE 1:**
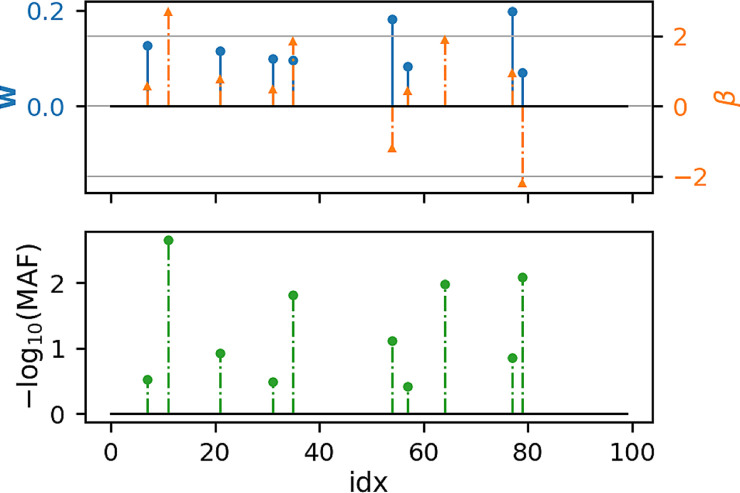
Recovered weights using the ecSKAT objective to find ***w*** when *y* is continuous. The *y*-axis is the magnitude of each weight, and the *x*-axis is the index of the true weight vector *β* and the found weights ***w***. The true weights *β* are sparse, and the marginal distribution has minor allele frequency (MAF), following a power law, with indices for which MAF is lower, typically leading to weights of large magnitude if they are non-zero. ***w*** is sparse with the non-zero indices falling in the set of non-zero indices of *β* but fails to learn large weights that correspond to extremely rare SNPs.

**FIGURE 2:**
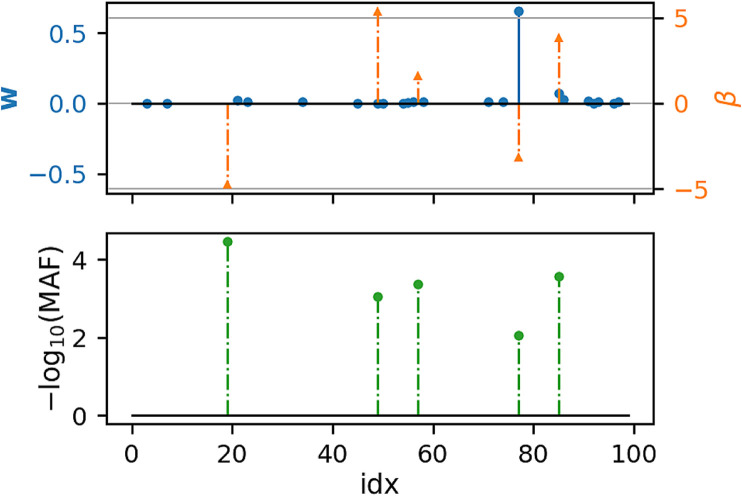
Recovered weights using the ecSKAT objective to find ***w*** when *y* is binary. The *y*-axis is the magnitude of each weight, and the *x*-axis is the index of the true weight vector *β* and the found weights ***w***. The true weights *β* are sparse, and the marginal distribution has minor allele frequency (MAF), following a power law, with indices for which the MAF is lower, typically leading to weights of large magnitude if they are non-zero. ***w*** is sparse with the non-zero indices overlapping with the set of non-zero indices of *β* but fails to learn large weights that correspond to extremely rare SNPs and has some small non-zero entries not overlapping with the non-zero indices of *β*.

Figures 1, 2 show that under idealized settings, ecSKAT manages to recover sensible weights, in particular, weights that add mass to semi-rare causal variants. Although this does not prove that the resulting test will perform well, it provides evidence that ecSKAT discards non-causal SNPs.

**FIGURE 3:**
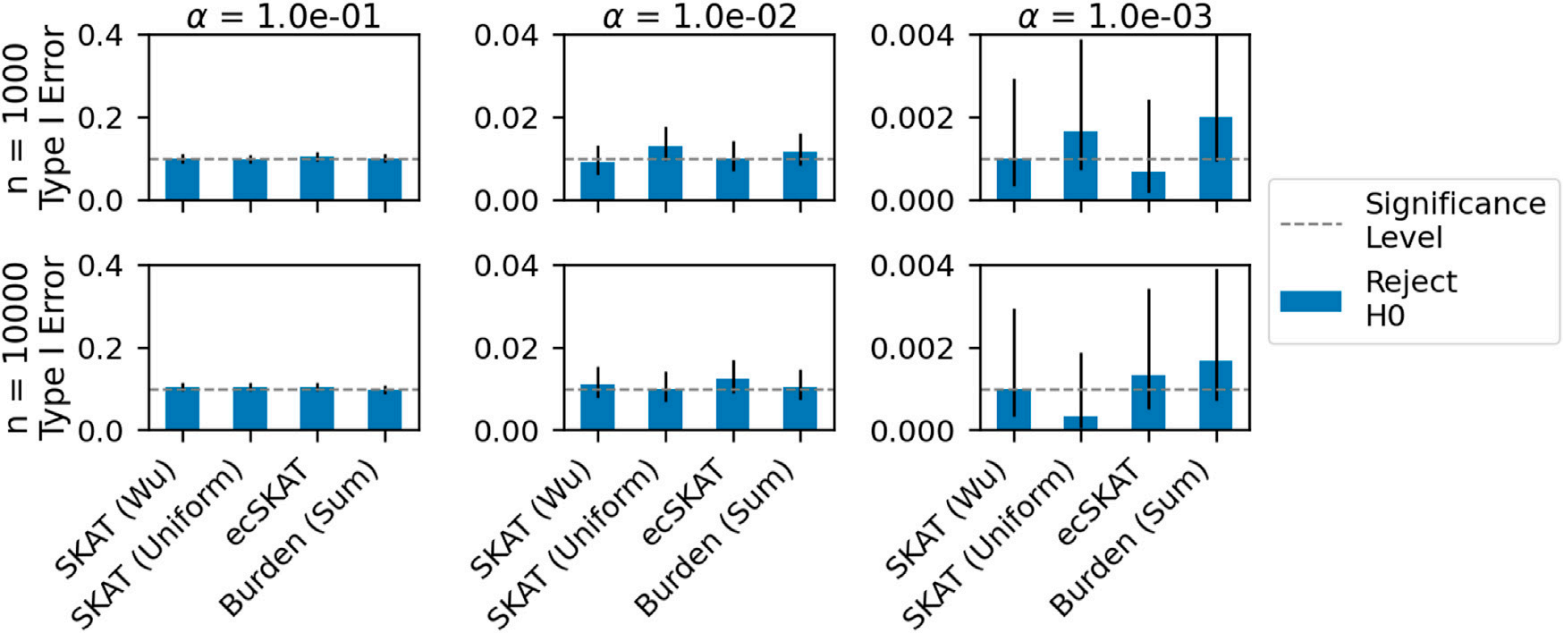
Type I error analysis for the correctly specified setting when *y* is continuous for all of the benchmarked algorithms (SKAT with weights of Wu et al. (2011), SKAT with uniform weights, our algorithm ecSKAT, and the burden test with sum aggregation). The columns are ordered by the pre-specified significance levels of 10^−1^,10^−2^,10^−3^, and the rows range from *n* =1000 to *n* =10000. We obtain the mean Type I error and 95% confidence intervals by repeating the setup 1,000 times and calculating the fraction of times that the algorithms choose to reject the null hypothesis.

In the Type I error experiment (Figure 3), we show how the Type I error behaves as a function of the size of the dataset under the correctly specified setting. We test this for two sample sizes, 1,000 and 1,0000, and for *α* = 10^−1^, 10^−2^, 10^−3^, and perform this experiment 1,000 times to get 95% confidence intervals. We see that all algorithms control the Type I error for big *α* but struggle for *α* = 10^−3^. However, it should be noted that for all algorithms in all plots, the current significance level falls within the confidence interval.

**FIGURE 4:**
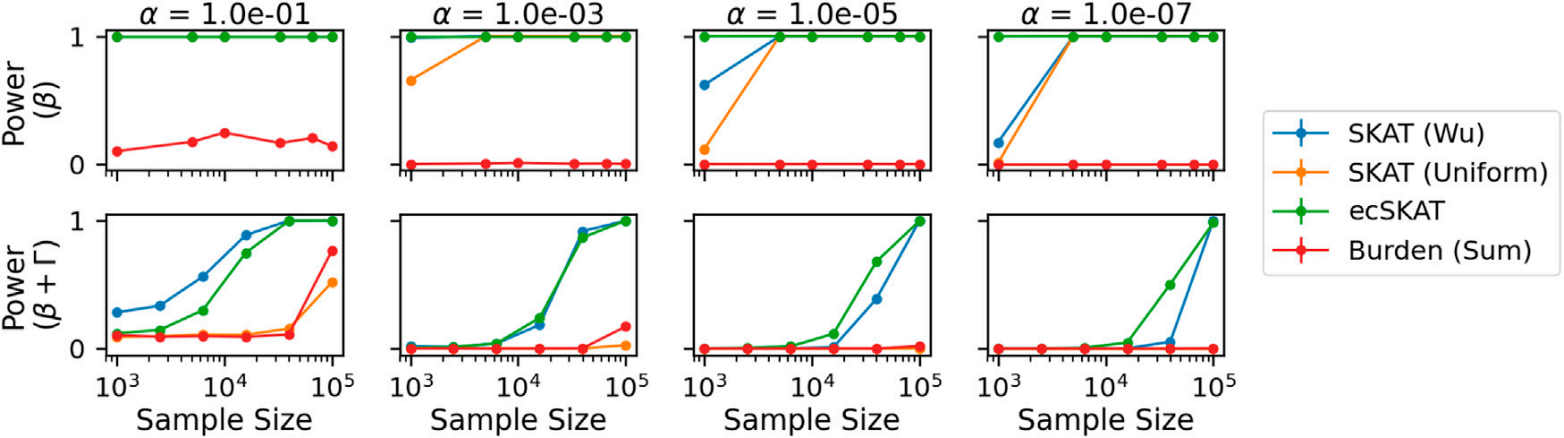
Power analysis when *y* is continuous for all benchmarked algorithms (SKAT with weights of Wu et al. (2011), SKAT with uniform weights, our algorithm ecSKAT, and the burden test with sum aggregation). The columns indicate different significance levels used *α* = 10^−1^, 10^−3^, 10^−5^, 10^−7^, and the rows specify different true functional relationships (no misspecification and misspecification with the additional covariance structure). We obtain the power mean and 95% confidence intervals by repeating the setup 1,000 times and calculating the fraction of times that the algorithms choose to correctly reject the null hypothesis.

Finally, for the power experiment (Figure 4), we look at the power of the correct (row 1) and misspecified cases (row 2) for different significance levels. As in the Type I error experiment, we repeat this experiment 1,000 times and calculate 95% confidence intervals. It should be noted that the confidence intervals are too small to be seen. For the correctly specified case (row 1), ecSKAT rejects the null hypothesis correctly for all plots, which can be seen by the straight line at 1 (maximum power). For a smaller *α* and large sample size, the other SKAT methods (*β*(1, 25), Uniform) also have maximum power, while the burden (Sum) test fails to perform well, probably because it assumes all weights to have the same sign, which is not true here. For the misspecified case (row 2), we see that only SKAT (*β*(1, 25)) and ecSKAT manage to perform well with their performance improving in the sample size and decreasing with smaller *α*. From this, we can see that ecSKAT performs best and SKAT variants perform well in terms of power for the correctly specified case, while only ecSKAT and SKAT (*β*(1, 25)) perform well in the misspecified case, probably due to the data-dependent nature of how they reweigh each genetic covariate.

### 3.2 Application to the UKBiobank data

We applied our proposed method to analyze the UK Biobank exome sequencing data. We tested associations of hand grip strength (quantitative trait) in 73,424 individuals and systemic lupus erythematosus (SLE) (binary trait) with 966 cases and 4,296 controls, adjusting for sex, age, and 10 principal components. We restricted our analysis to the predicted loss-of-function (LoF; i.e., essential splice site changes, stop codon gain, or frameshifts) (MacArthur et al., 2012)) variants with MAF <0.01. In addition to testing for an association via ecSKAT, we also applied the weighted sum burden test, weighted max burden test, and SKAT with a weighting of β(1, 25)-pdf of the MAF. For handgrip strength, we analyzed variants in *TPTEP2-CSNK1E* and *ZDHHC8* (Karczewski et al., 2022), which were reported to be associated with the phenotype in Genebass. For SLE, we uncovered the association of aggregation of LoF in gene *PCSK9,* which was not reported in Genebass but was shown to be associated with disease activity in SLE (Frostegard et al., 2020). The underlying cause could be that oxidized LDL promotes DC activation, which depends on *PCSK9*, with a higher effect among SLE patients. *PCSK9* could play an unexpected immunological role in SLE. Our proposed ecSKAT was the most powerful test for both quantitative and binary traits and has much smaller *p*-values than the burden test and SKAT for the genes that we tested (Tables 1, 2).

**TABLE 1:** Association test for hand grip strength (*p*-value).

		Method			
Gene	Category	Burden (sum)	Burden (max)	SKAT	ecSKAT
*TPTEP2-CSNK1E*	LoF	5.14 × 10^−2^	5.14 × 10^−2^	4.73 × 10^−2^	3.08 × 10^−2^
*ZDHHC8*	LoF	4.91 × 10^−2^	4.91 × 10^−2^	4.85 × 10^−2^	3.76 × 10^−2^

**TABLE 2:** Association test for systemic lupus erythematosus (*p*-value).

		Method			
Gene	Category	Burden (sum)	Burden (max)	SKAT	ecSKAT
*PCSK9*	LoF	1.4 × 10^−5^	2.5 × 10^−5^	1.0 × 10^−5^	0.8 × 10^−5^
*CSNK2A1*	LoF	2.47 × 10^−2^	2.47 × 10^−2^	2.45 × 10^−2^	2.31 × 10^−2^

## 4 Discussion

This study generalised the cSKAT formulation to general GLM models with non-genetic covariates and showed that this formulation, while being considerably more general and applicable in practice as compared to the linear model, the no covariate setting of Posner et al. (2020) still allows for finding the optimal weights through a QP, thus being equally computationally complex to the simpler setting. Our theoretical and methodological contributions are threefold.

1. We showed that the weighted annotation method of Posner et al. (2020) can be formulated as an instance of the SKAT setting where we first apply a linear feature map to the genetic covariates.
2. We completed the analysis of cSKAT for the case of an arbitrary GLM model when the covariates are non-zero, showing that the objective can be solved using a similar QP procedure as the original cSKAT algorithm, retaining the same computational complexity.
3. Finally, we showed that a slight modification of the cSKAT objective is related to an upper bound on the *p*-value as a function of ***w*** and that this bound is tight as the objective goes to infinity, indicating that cSKAT is a principled objective since it relates well to the objective of the study (the actual *p*-value).

Simulation studies showed that ecSKAT can recover sensible weights and achieve higher power across different sample sizes and misspecification settings. In real data analysis, we applied the method to both the binary (SLE) and quantitative (hand grip strength) traits in the UKBiobank cohort. Compared to the burden test and SKAT method, ecSKAT gives a slightly lower *p*-value for the genes tested in both quantitative and binary traits.

In the future, we would like to theoretically analyze the power in terms of the true value ***w*** * and the size of the training and validation sets. Furthermore, given a fixed dataset size *n*, we would like to analyze the optimal training set size, which would be of interest in practice. Finally, we would like to perform more large-scale experiments, in particular, on the newly released 500-k WES cohort of UKBiobank (Backman et al., 2021; Szustakowski et al., 2021).

## Data Availability

The data analyzed in this study are subjected to the following licenses/restrictions: The UK Biobank resource is available to *bona fide* researchers for health-related research in the public interest and can be accessed via the Access Management System. Requests to access these datasets should be directed to UKBiobank.

## Footnotes

1 The value is given by the number of minor variants of the SNP at the marker, and other schemes can trivially be handled.

2
*N*(*μ*, ***V***) is the Gaussian distribution with mean *μ* and covariance matrix ***V***.

3 For a vector $x\in R^{d}$ , diag(***x***) is the *n* × *n* diagonal matrix with ***x*** on the diagonal.

4 Explicitly, ***w*** ∈ △*p* is equivalent for any *i* ∈ {1, *…*, *p*}, ***w*** *i* ≥ 0, and $\sum_{i=1}^{p}w_{i}=1$ .
