# Supplementary material for: Learning the kernel for rare variant genetic association test
Source: Front Genet. 2023 Oct 9;14:1245238. doi: 10.3389/fgene.2023.1245238 (PMC10598548; doi:10.3389/fgene.2023.1245238)
Supplement: Supplementary file 1 [file Presentation1.pdf]

1 APPENDIX

1.1 Notation

For an integer n let $[n] = \{1, \dots, n\}$. Let $\mathbb{R}^n$ be the space of n-dimensional real vectors identified with column vectors, $\mathbb{R}^{n\times m}$ the space of $n \times m$ matrices. Furthermore let S^n be the space of $n \times n$ dimensional real symmetric matrices and S^n_+, S^n_{++} positive-semidefinite and positive-definite matrices $n \times n$ dimensional respectively. Let $\mathbb{R}^n_+, \mathbb{R}^n_{++}$ be the space of n-dimensional real vectors with all components being non-negative and positive respectively. Let $\triangle_n$ be the set of vectors in $\mathbb{R}^n_+$ summing to one (the set of probability vectors). We let boldface denote matrices / vectors, such as $\boldsymbol{X}$ and let $\boldsymbol{X}^\mathsf{T}$ be the transpose of $\boldsymbol{X}$. Let $\mathbf{1}_n, \mathbf{0}_n$ be the n-dimensional vectors of all ones and zeros and let $\mathbf{1}_{n,m}, \mathbf{0}_{n,m}$ be the $n \times m$-dimensional matrices of ones and zeros respectively. We will drop the subscript when the dimensions are clear from context. For two vectors $\boldsymbol{x}, \boldsymbol{y} \in \mathbb{R}^n$ let $\langle \boldsymbol{x}, \boldsymbol{y} \rangle = \boldsymbol{x}^\mathsf{T}\boldsymbol{y} = \sum_{i=1}^n x_i y_i$ and let $\|\boldsymbol{x}\|_p = (\sum_{i=1}^n x_i^p)^{1/p}$ which is a true norm when $p \geq 1$, with $\|\boldsymbol{x}\|_\infty = \max\left((|\boldsymbol{x}_i|)_{i=1}^n\right)$. For two matrices $\boldsymbol{X}, \boldsymbol{Y} \in \mathbb{R}^{n\times m}$ let $\langle \boldsymbol{X}, \boldsymbol{Y} \rangle = \sum_{i,j}^{n,m} \boldsymbol{X}_{ij}\boldsymbol{Y}_{ij}$ be the Frobenius inner product and $\|\boldsymbol{X}\|_{FB}$ be the induced norm, the Frobenius norm and let $\boldsymbol{X} \odot \boldsymbol{Y}$ be the hadamard product, $(\boldsymbol{X} \odot \boldsymbol{Y})_{i,j} = \boldsymbol{X}_{i,j}\boldsymbol{Y}_{i,j}$. Let $\boldsymbol{X} \in \mathbb{R}^{n\times n}$ then the trace is define to be $\mathrm{Tr}(\boldsymbol{X}) = \sum_{i=1}^n \boldsymbol{X}_{ii}$ and if $\boldsymbol{X}$ can be eigendecomposed with eigenvalues $(\lambda_i)_{i=1}^n$ then $\mathrm{Tr}(\boldsymbol{X}) = \sum_{i=1}^n \lambda_i$ and $\det(\boldsymbol{X}) = \prod_{i=1}^n \lambda_i$. Let $\boldsymbol{X} \in \mathbb{R}^{n\times m}$ and $I = \{I_j : j = 1, \dots, |I|\}$ be a partition of $[n]$ and $J = \{J_j : j = 1, \dots, |J|\}$ be a partition of $[m]$. We let $\boldsymbol{X}_{I_j}$ be the rows corresponding to I_j stacked as an $\mathbb{R}^{|I_j|\times m}$ matrix and $\boldsymbol{X}^{J_j}$ be the columns corresponding to J_j stacked as an $\mathbb{R}^{n\times|J_j|}$ matrix. Let $\mathcal{H}$ denote an RKHS and K the corresponding kernel.

We will use capital letter to denote a random variable and the lower-case for the observation, X, x. Let $\mathbb{I}(E)$ denote the indicator variable of the event E. For a family of distributions ρ_θ parameterized by $\theta \in \Theta$ with a pdf we write $p_\rho(y; \theta)$ for the value of the pdf of ρ_θ at y. Below is a table of distributions we will use together with some information about these distributions. Let $\mathrm{B}(\alpha) = \prod_{i=1}^n \Gamma(\alpha_i)/\Gamma(\sum_{i=1}^n \alpha_i)$ be the beta-function.

1.2 On a flaw in the cSKAT derivation for the extended setting

In this section we detail a flaw in the derivation of cSKAT extended to the general case in (Posner et al., 2020, Appendix A.1). We state it in terms of our notation.

The flaw can be found in (Posner et al., 2020, Eq. 11 and Eq. 12) and the derivation is found in (Posner et al., 2020, A.1), which is due to not using the right denominator $\|\boldsymbol{\lambda}_0\|_2$ in the objective when going beyond the continuous case and / or having non-genetic covariates (note that we cancel out the $\hat{\phi}_0$ terms since they occur in both the numerator and denominator). As stated on (Posner et al., 2020, p. 4) the $\hat{\phi}_0 \cdot \boldsymbol{\lambda}_0 = \mathrm{eig}(\boldsymbol{P}_0^{1/2}\boldsymbol{K}\boldsymbol{P}_0^{1/2})$. For the continuous case with no non-genetic covariates, $\boldsymbol{P}_0$ can be shown to be the centering matrix, and assuming that kernel matrix $\boldsymbol{K}$ is centered, the objective reduces to $\boldsymbol{y}^\mathsf{T}\boldsymbol{K}\boldsymbol{y}/\|\boldsymbol{K}\|_{FB}$ since $\|\boldsymbol{\lambda}_0\|_2 = \|\boldsymbol{K}\|_{FB}$ in this case as 1) $\boldsymbol{K}$ is centered and so $\boldsymbol{P}_0^{1/2}\boldsymbol{K}\boldsymbol{P}_0^{1/2} = \boldsymbol{K}$ and 2) $\boldsymbol{P}_0$ is the centering matrix (which is only true in this particular case). However, when including the $\boldsymbol{V}$ or $\boldsymbol{X}$ terms in $\boldsymbol{P}_0$, $\boldsymbol{P}_0$ is no longer the centering matrix and so the numerator and denominator need to change as follows; 1) $\boldsymbol{y}^T\boldsymbol{K}\boldsymbol{y}$ turns into $\boldsymbol{r}^T\boldsymbol{K}\boldsymbol{r}$ where $\boldsymbol{r}$ is the residual vector under the null-hypothesis and 2) $\|\boldsymbol{\lambda}_0\|_2$ is no longer equal to $\|\boldsymbol{K}\|_{FB}$ but to $\|\boldsymbol{P}_0^{1/2}\boldsymbol{K}\boldsymbol{P}_0^{1/2}\|_{FB}$. Point 2) is flawed in their analysis since they use $\|\boldsymbol{K}\|_{FB}$ instead of $\|\boldsymbol{P}_0^{1/2}\boldsymbol{K}\boldsymbol{P}_0^{1/2}\|_{FB}$ which leads to the wrong objective.

1.3 Theorems

THEOREM 1. *Assume a weighted linear kernel $K_{\boldsymbol{w}}$. Given a dataset $D = (\boldsymbol{X}, \boldsymbol{G}, \boldsymbol{y})$ and a GLM model $\eta(\mu(x, g)) = \alpha_0 + \alpha^\top x + \beta^\top g$ giving rise to residuals $\boldsymbol{r} = \boldsymbol{y} - \hat{\mu}_0$, variance matrix $\boldsymbol{V} = \operatorname{diag}(\boldsymbol{v})$, null projection matrix $\boldsymbol{P}_0$ and matrix $\boldsymbol{A} = \boldsymbol{P}_0^{1/2}\boldsymbol{K}\boldsymbol{P}_0^{1/2}/\hat{\phi}_0$ where $\boldsymbol{K} = \boldsymbol{G}\boldsymbol{W}\boldsymbol{G}^\top$ and $\hat{\phi}_0$ is the null maximum likelihood deviance parameter. Let $\boldsymbol{B} = \boldsymbol{G}^\top(\boldsymbol{V} - \boldsymbol{V}\boldsymbol{X}(\boldsymbol{X}^\top\boldsymbol{V}\boldsymbol{X})^{-1}\boldsymbol{X}^\top\boldsymbol{V})\boldsymbol{G}$, then*

$$J(\boldsymbol{w}) = \frac{\boldsymbol{w}^\top \boldsymbol{s}}{\|\boldsymbol{w}\|_{\boldsymbol{B}\odot\boldsymbol{B}}} \tag{1}$$

and

$$\boldsymbol{w}^* = \arg\max_{\boldsymbol{w}\in\triangle_p} J(\boldsymbol{w}) \propto \arg\min_{\boldsymbol{z}\geq 0} \boldsymbol{z}^\top(\boldsymbol{B}\odot\boldsymbol{B})\boldsymbol{z} - 2\boldsymbol{z}^\top\boldsymbol{s}. \tag{2}$$

PROOF. Note that $J(\boldsymbol{w}) = \boldsymbol{r}^\top\boldsymbol{K}\boldsymbol{r}/\|\operatorname{eig}(\boldsymbol{P}_0^{1/2}\boldsymbol{K}\boldsymbol{P}_0^{1/2})\|_2$ cancelling out $\hat{\phi}_0$. We first simplify the numerator and denominator, starting with the numerator. We will use the identity Horn and Johnson (2012)

$$\operatorname{Tr}(\operatorname{diag}(\boldsymbol{v})\boldsymbol{M}\operatorname{diag}(\boldsymbol{u})\boldsymbol{N}^\top) = \boldsymbol{v}^\top(\boldsymbol{M}\odot\boldsymbol{N})\boldsymbol{u} \tag{3}$$

at several points in the proof.

First note that

$$\boldsymbol{r}^\top\boldsymbol{K}\boldsymbol{r} = \boldsymbol{r}^\top\boldsymbol{G}\boldsymbol{W}\boldsymbol{G}^\top\boldsymbol{r} = \operatorname{Tr}(\operatorname{diag}(\boldsymbol{w})\boldsymbol{G}^\top\boldsymbol{r}\operatorname{diag}(1)(\boldsymbol{G}^\top\boldsymbol{r})^\top) \tag{4}$$

which by (3) is equal to $\boldsymbol{w}^\top(\boldsymbol{G}^\top\boldsymbol{r}\odot\boldsymbol{G}^\top\boldsymbol{r})1 = \boldsymbol{w}^\top\boldsymbol{s}$. For the denominator we have

$$\|\operatorname{eig}(\boldsymbol{P}_0^{1/2}\boldsymbol{K}\boldsymbol{P}_0^{1/2})\|_2^2 = \operatorname{Tr}((\boldsymbol{P}_0^{1/2}\boldsymbol{K}\boldsymbol{P}_0^{1/2})^2) \tag{5}$$

$$= \operatorname{Tr}(\boldsymbol{P}_0^{1/2}\boldsymbol{K}\boldsymbol{P}_0\boldsymbol{K}\boldsymbol{P}_0^{1/2}) \tag{6}$$

$$= \operatorname{Tr}(\boldsymbol{K}\boldsymbol{P}_0\boldsymbol{K}\boldsymbol{P}_0) \tag{7}$$

$$= \operatorname{Tr}(\boldsymbol{G}\boldsymbol{W}\boldsymbol{G}^\top\boldsymbol{P}_0\boldsymbol{G}\boldsymbol{W}\boldsymbol{G}^\top\boldsymbol{P}_0) \tag{8}$$

$$= \operatorname{Tr}(\boldsymbol{W}\boldsymbol{B}\boldsymbol{W}\boldsymbol{B}), \tag{9}$$

which, since $\boldsymbol{B}\in S_+^p$, is of the form required by (3) meaning that

$$\|\operatorname{eig}(\boldsymbol{P}_0^{1/2}\boldsymbol{K}\boldsymbol{P}_0^{1/2})\|_2 = \sqrt{\boldsymbol{w}^\top(\boldsymbol{B}\odot\boldsymbol{B})\boldsymbol{w}} = \|\boldsymbol{w}\|_{\boldsymbol{B}\odot\boldsymbol{B}}, \tag{10}$$

where since $\boldsymbol{B}$ is positive semi-definite (or positive definite) so is $\boldsymbol{B}\odot\boldsymbol{B}$ Styan (1973).

Combining the above, we have that

$$J(\boldsymbol{w}) = \frac{\boldsymbol{w}^\top\boldsymbol{s}}{\|\boldsymbol{w}\|_{\boldsymbol{B}\odot\boldsymbol{B}}}. \tag{11}$$

Finally, it can be seen that the results of Cortes et al. (2012) still applies and finding $\boldsymbol{w}^*$ is equivalent to solving the Quadratic Programme

$$\boldsymbol{z}^* = \arg\min_{\boldsymbol{z}\geq 0} \boldsymbol{z}^\top(\boldsymbol{B}\odot\boldsymbol{B})\boldsymbol{z} - 2\boldsymbol{z}^\top\boldsymbol{s} \tag{12}$$

and letting $\boldsymbol{w}^* = \boldsymbol{z}^*/\|\boldsymbol{z}^*\|_1$.

DEFINITION 1 (Sub-exponential random variable, Wainwright (2019) Definition 2.7). *A random variable X with mean $\mu = \mathbb{E}X$ is* sub-exponential *if there are non-negative parameters (ν, α) such that*

$$\mathbb{E}[\exp(\lambda(X - \mu))] \leq \exp(\nu^2\lambda^2/2) \quad \textit{for all } |\lambda| \leq 1/\alpha, \tag{13}$$

and we write this as $X \in \mathrm{SE}(\nu, \alpha)$.

LEMMA 2 (Scaled sub-exponential variable is sub-exponential). *Let X be sub-exponential with parameters (ν, α), then for any $c > 0$, cX is sub-exponential with parameters $(c\nu, c\alpha)$.*

PROOF. Let $\mu = \mathbb{E}X$ and let $Y = cX$. Then $\mathbb{E}Y = c\mu$. Since X is sub-exponential it satisfies

$$\mathbb{E}[\exp(\lambda(X - \mu))] \leq \exp(\nu^2\lambda^2/2) \quad \text{for all } |\lambda| \leq 1/\alpha.$$

Now,

$$\mathbb{E}[\exp(\lambda(Y - \mathbb{E}Y))] = \mathbb{E}[\exp(c\lambda(X - \mu)] \leq \exp(c^2\nu^2\lambda^2/2) \quad \text{for all } |c\lambda| \leq 1/\alpha, \tag{14}$$

since $|c\lambda| \leq 1/\alpha \iff |\lambda| \leq 1/c\alpha$ we are done.

LEMMA 3 (χ_1^2 is sub-exponential, Wainwright (2019) Example 2.8). *Let X be a χ_1^2 random variable, then X is sub-exponential with parameters $(2, 4)$.*

LEMMA 4 (Linear combination of sub-exponential random variables is sub-exponential, Wainwright (2019) p. 29). *Let $(X_i)_{i=1}^n$ be a sequence of sub-exponential random variables with parameters $(\nu_i, \alpha_i)_{i=1}^n$ and means $(\mu_i)_{i=1}^n$. Then $X = \sum_{i=1}^n (X_i - \mu_i)$ is a sub-exponential random variable with parameters (ν_*, α_*), with $\nu_* = \sqrt{\sum_{i=1}^n \nu_i^2} = \|\nu\|_2$ and $\alpha_* = \max_{i=1}(\alpha_i) = \|\alpha\|_\infty$ where $\nu = (\nu_i)_{i=1}^n$ and $\alpha = (\alpha_i)_{i=1}^n$.*

PROPOSITION 5 (Sub-exponential Tail Bound, Wainwright (2019) Prop. 2.9). *Suppose that X is sub-exponential with parameters (ν, α) with mean μ, then for any $t \geq 0$*

$$\Pr(X - \mu \geq t) \leq \begin{cases} \exp\left(-\frac{t^2}{2\nu^2}\right), & \textit{if } 0 \leq t \leq \frac{\nu^2}{\alpha} \\ \exp\left(-\frac{t}{2\alpha}\right), & \textit{if } t > \frac{\nu^2}{\alpha} \end{cases} = \exp\left(-\frac{1}{2}\min\left(\frac{t^2}{\nu^2}, \frac{t}{\alpha}\right)\right) \tag{15}$$

THEOREM 6 (cSKAT objective upper bounds the p-value). *Assume a weighted linear kernel $K_{\boldsymbol{w}}$. Given a dataset $D = (\boldsymbol{X}, \boldsymbol{G}, \boldsymbol{y})$ and a GLM model $\eta(\mu(x, g)) = \alpha_0 + \alpha^\mathsf{T} x + \beta^\mathsf{T} g$ giving rise to residuals $\boldsymbol{r} = \boldsymbol{y} - \hat{\mu}_0$, variance matrix $\boldsymbol{V} = \mathrm{diag}(\boldsymbol{v})$, null projection matrix $\boldsymbol{P}_0$ and matrix $\widetilde{\boldsymbol{A}} = \boldsymbol{P}_0^{1/2}\boldsymbol{K}\boldsymbol{P}_0^{1/2}$, let*

$\widetilde{\lambda}(\boldsymbol{w})_0 = \mathrm{eig}(\widetilde{\boldsymbol{A}})$, $\ell_p = \|\widetilde{\boldsymbol{\lambda}}_0(\boldsymbol{w})\|_p$, *where* $p \in [1, \infty]$, *and* $\widetilde{q}(\boldsymbol{w}) = \boldsymbol{r}^\mathsf{T}\boldsymbol{G}\boldsymbol{W}\boldsymbol{G}^\mathsf{T}\boldsymbol{r}$, *then*

$$\mathrm{p}_0(q(\boldsymbol{w})) \leq \begin{cases} \exp\left(-\frac{1}{8}\left(\frac{\widetilde{q}(\boldsymbol{w})-\ell_1}{\ell_2}\right)^2\right), & \textit{if } \widetilde{q}(\boldsymbol{w}) \in [\ell_1, \frac{\ell_2^2}{\ell_\infty} + \ell_1] \\ \exp\left(-\frac{1}{8}\left(\frac{\widetilde{q}(\boldsymbol{w})-\ell_1}{\ell_\infty}\right)\right), & \textit{if } \widetilde{q}(\boldsymbol{w}) \geq \frac{\ell_2^2}{\ell_\infty} + \ell_1. \end{cases} \tag{16}$$

Let $\boldsymbol{B} = \boldsymbol{G}^\mathsf{T}(\boldsymbol{V} - \boldsymbol{V}\boldsymbol{X}(\boldsymbol{X}^\mathsf{T}\boldsymbol{V}\boldsymbol{X})^{-1}\boldsymbol{X}^\mathsf{T}\boldsymbol{V})\boldsymbol{G}$, *and* $\boldsymbol{b} = \mathrm{diag}(\boldsymbol{B})$. *If* $J(\boldsymbol{w}) = \frac{\boldsymbol{w}^\mathsf{T}(\boldsymbol{s}-\boldsymbol{b})}{\|\boldsymbol{w}\|_{\boldsymbol{B}\odot\boldsymbol{B}}}$ *and* $\boldsymbol{w}^\mathsf{T}\boldsymbol{s} \geq \ell_1$. *If* $\widetilde{q}(\boldsymbol{w}) \geq \ell_1$ *then*

$$\mathrm{p}_0(q(\boldsymbol{w})) \leq \exp\left(-\frac{1}{8}\min\left(J(\boldsymbol{w}), J(\boldsymbol{w})^2\right)\right). \tag{17}$$

PROOF. Let $\widetilde{Q}(\boldsymbol{w}) = \sum_{i=1}\widetilde{\boldsymbol{\lambda}}_0(\boldsymbol{w})_i Y_i = \sum_i X_i$ where $(Y_i)_{i=1}$ are iid and distributed as $Y_1 \sim \chi_1^2$ and thus $(X_i)_i$ are independent and $X_i \sim \widetilde{\boldsymbol{\lambda}}_0(\boldsymbol{w})_i\chi_1^2$.

The p-value of the model is given by

$$\mathrm{p}_0(q(\boldsymbol{w})) = \Pr(Q_{\mathsf{SKAT}}(\boldsymbol{w}) \geq q(\boldsymbol{w})) = \Pr(\widetilde{Q}(\boldsymbol{w}) \geq \widetilde{q}(\boldsymbol{w})). \tag{18}$$

We note the following,

1. $\tilde{\mu} = \mathbb{E}\widetilde{Q}(\boldsymbol{w}) = \sum_i \widetilde{\boldsymbol{\lambda}}_0(\boldsymbol{w})_i\mathbb{E}\chi_1^2 = \|\widetilde{\boldsymbol{\lambda}}_0(\boldsymbol{w})\|_1$,
2. by Lemma 3 we know that $\chi_1^2 \sim \mathrm{SE}(2, 4)$ and applying Lemma 2 $X_i \in \mathrm{SE}(2\widetilde{\boldsymbol{\lambda}}_0(\boldsymbol{w})_i, 4\widetilde{\boldsymbol{\lambda}}_0(\boldsymbol{w})_i)$ and finally applying Lemma 4 we have that $\widetilde{Q}(\boldsymbol{w}) \in \mathrm{SE}(\nu_*, \alpha_*)$ where $\nu_* = \|\widetilde{\boldsymbol{\lambda}}_0(\boldsymbol{w})\|_2$ and $\alpha_* = \|\widetilde{\boldsymbol{\lambda}}_0(\boldsymbol{w})\|_\infty$.

Call $t = \widetilde{q}(\boldsymbol{w}) - \tilde{\mu}$, and denote by $\ell_p = \|\widetilde{\boldsymbol{\lambda}}_0(\boldsymbol{w})\|_p$, where $p \in [1, \infty]$, then applying Prop. 5 we have that

$$\Pr(\widetilde{Q}(\boldsymbol{w}) \geq \widetilde{q}(\boldsymbol{w})) = \Pr(\widetilde{Q}(\boldsymbol{w}) - \tilde{\mu} \geq t) \leq \begin{cases} \exp\left(-\frac{t^2}{2\nu_*^2}\right), & \text{if } 0 \leq t \leq \frac{\nu_*^2}{\alpha_*} \\ \exp\left(-\frac{t}{2\alpha_*}\right), & \text{if } t > \frac{\nu_*^2}{\alpha_*}. \end{cases} \tag{19}$$

Writing out (19) explicitly, we see that the first case states that for $\widetilde{q}(\boldsymbol{w}) \in [\ell_1, \frac{\ell_2^2}{\ell_\infty} + \ell_1]$, the p-value is upper bounded by $\exp(-\frac{1}{8}(\frac{\widetilde{q}(\boldsymbol{w})-\ell_1}{\ell_2})^2)$ while the second case states that for $\widetilde{q}(\boldsymbol{w}) > \frac{\ell_2^2}{\ell_\infty} + \ell_1$ the p-value is upper bounded by $\exp(-\frac{1}{8}(\frac{\widetilde{q}(\boldsymbol{w})-\ell_1}{\ell_\infty}))$.

Then second statement is proved as follows. Since $\widetilde{q}(\boldsymbol{w}) = \boldsymbol{w}^\mathsf{T}\boldsymbol{s}$ and similarly $\ell_1 = \mathrm{Tr}(\boldsymbol{P}_0^{1/2}\boldsymbol{G}\boldsymbol{W}\boldsymbol{G}^\mathsf{T}\boldsymbol{P}_0^{1/2}) = \mathrm{Tr}(\boldsymbol{W}\boldsymbol{B}) = \boldsymbol{w}^\mathsf{T}\boldsymbol{b}$, both following by similar arguments as in (4), so that $\widetilde{q}(\boldsymbol{w}) - \ell_1 = \boldsymbol{w}^\mathsf{T}(\boldsymbol{s} - \boldsymbol{b})$. Following (10) we have that $\ell_2 = \|\boldsymbol{w}\|_{\boldsymbol{B}\odot\boldsymbol{B}}$ so

$$\frac{\widetilde{q}(\boldsymbol{w}) - \ell_1}{\ell_2} = \frac{\boldsymbol{w}^\mathsf{T}(\boldsymbol{s} - \boldsymbol{b})}{\|\boldsymbol{w}\|_{\boldsymbol{B}\odot\boldsymbol{B}}} = J(\boldsymbol{w}). \tag{20}$$

Finally, since $\ell_\infty \leq \ell_2$ we have that $\frac{\widetilde{q}(\boldsymbol{w})-\ell_1}{\ell_2} \leq \frac{\widetilde{q}(\boldsymbol{w})-\ell_1}{\ell_\infty}$ which means that for $\widetilde{q}(\boldsymbol{w}) > \frac{\ell_2^2}{\ell_\infty} + \ell_1$ and so

$$\exp\left(-\frac{1}{8}\left(\frac{\widetilde{q}(\boldsymbol{w}) - \ell_1}{\ell_\infty}\right)\right) \leq \exp\left(-\frac{1}{8}\left(\frac{\widetilde{q}(\boldsymbol{w}) - \ell_1}{\ell_2}\right)\right) = \exp\left(-\frac{1}{8}J(\boldsymbol{w})\right), \tag{21}$$

which in total shows that if $\widetilde{q}(\boldsymbol{w}) > \ell_1$ then

$$\mathrm{p}_0(q(\boldsymbol{w})) \leq \exp\left(-\frac{1}{8}\min\left(J(\boldsymbol{w}), J(\boldsymbol{w})^2\right)\right). \tag{22}$$

1.4 Experimental Setup

For each of the hypothesis testing settings we use the UKBB WES dataset of the gene PARK7 resulting in 200'643 patients or datapoints. The PARK7 gene has 462 variants. For each experiment we sample without replacement n number of patients, where n is dependent on the situation. This means that for each experiment we run, we get a new genetic and non-genetic covariance matrix of n rows and 462 columns for the genetic matrix and 12 columns for the non-genetic matrix since we keep the columns `sex, age` and the first 10 principal components from the full genetic matrix on the whole UKBB WES dataset.

Each setting is specified and generated by specifying the `causal_ratio` (which we set to 0.1) which is ratio of causal variants in the gene, which is sampled with replacement according to the probability vector generated from taking all of the MAF's and mapping them through $f(x) = x^{-0.5}$ and finally normalize so that it sums to one. In this way we satisfy the common assumption that rarer variants are more often causal. For the interaction term (if it is used) we have a parameter `interaction_ratio` (which we set to 1.0 so all causal variants interact) which specify how many of the causal variants interact with other variants and we also have a `misspecification_factor` which scales the final interaction matrix Γ so that $\|\beta\|/\|\Gamma\|_2 =$ `misspecification_factor` which we set to 1.0, which means we are severely misspecified. For each causal variant, the beta coefficient of each causal variant SNP at index j is given by $-\log_{10}(\mathrm{MAF}_j)$ where MAF_j is calculated from the full dataset, and the sign is flipped according to a probability `beta_random_sign_flip_prob`. Finally, we use a link function to map from the mean to the sampling of the output. For continuous output, this is just the identity link and for binary this is the Bernoulli link function. We generate α, α_0 from a standard multivariate Gaussian and are then normalized to have norm 1.

For the interaction term, use pick a subset of the causal variants at random until `interaction_ratio` has been chosen at random and each such variant interacts at random with one of the other variants in the gene except for itself. The interaction term is added to the previously used α_0, α, β and so that the misspecified case does not change the linear terms used previously, but only add the interaction term.

For the continuous setting which corresponds to a Gaussian model, we set the noise variance to be $\sigma^2 = 4$ which corresponds to an h^2 (heritability) coefficient of 0.2 in this case.

As done in previous works (e.g. Posner et al. (2020)) we center all of the kernel matrices.

REFERENCES

Cortes, C., Mohri, M., and Rostamizadeh, A. (2012). Algorithms for learning kernels based on centered alignment. *The Journal of Machine Learning Research* 13, 795–828. doi:10.5555/2503308.2188413

Horn, R. A. and Johnson, C. R. (2012). *Matrix analysis* (Cambridge university press)

Posner, D. C., Lin, H., Meigs, J. B., Kolaczyk, E. D., and Dupuis, J. (2020). Convex combination sequence kernel association test for rare-variant studies. *Genetic epidemiology* 44, 352–367

Styan, G. P. (1973). Hadamard products and multivariate statistical analysis. *Linear algebra and its applications* 6, 217–240

Wainwright, M. J. (2019). *High-dimensional statistics: A non-asymptotic viewpoint*, vol. 48 (Cambridge University Press)
